# DDR1 Targeting HOXA6 Facilitates Bladder Cancer Progression via Inhibiting Ferroptosis

**DOI:** 10.1111/jcmm.70410

**Published:** 2025-03-19

**Authors:** Xin Xie, Hongchao He, Ning Zhang, Xiaojing Wang, Wenbin Rui, Danfeng Xu, Yu Zhu, Ming Tian, Wei He

**Affiliations:** ^1^ Department of Urology, Ruijin Hospital Shanghai Jiao Tong University School of Medicine Shanghai China; ^2^ Department of Burn, Ruijin Hospital Shanghai Jiao Tong University School of Medicine Shanghai China

**Keywords:** bladder cancer, DDR1, ferroptosis, HOXA6, progression

## Abstract

Ferroptosis is an important factor affecting the progression of bladder cancer (BC). Previous studies have confirmed that discoidin domain receptor 1 (DDR1) promotes BC progression. However, the regulatory mechanisms of BC ferroptosis are largely unknown. Therefore, this study aimed to investigate the regulatory effects of DDR1 on BC cell ferroptosis. Ferroptosis‐sensitive and ‐resistant BC cells were screened, and reverse‐transcription quantitative PCR and western blotting were used to determine the expression of DDR1 in BC cells. In vitro and in vivo assays were performed to analyse the mechanisms of DDR1 in BC ferroptosis. The ferroptosis inducer erastin inhibited DDR1 expression in TCCSUP cells. The ferroptosis inhibitor ferrostatin‐1 inhibited BC cell death caused by DDR1 knockdown. DDR1 increased glutathione, glutathione peroxidase 4 and solute carrier family 7 member 11 expression, while decreasing malondialdehyde and Fe^2+^ levels and acyl‐CoA synthetase long‐chain family member 4 levels and inhibiting epithelial mesenchymal transition and neurofibromin 2‐yes‐associated protein. These effects were abrogated by the knockdown of homeobox A6 (HOXA6). DDR1 targeting of HOXA6 facilitated BC growth and inhibited BC ferroptosis in vivo. DDR1 promotes BC progression by inhibiting ferroptosis and targeting HOXA6. Thus, DDR1 may serve as a potential therapeutic target for BC.

## 1 Background

Bladder cancer (BC) poses a serious threat to human health [1]. BC can be categorised as epithelial, squamous cell, or undifferentiated carcinoma or adenocarcinoma based on tissue origin or non‐muscle–invasive BC and muscle‐invasive BC based on disease pattern [2]. Globally, BC incidence ranks 9th among all cancers; its mortality rate ranks 13th among all cancer‐related deaths [3]. Despite advances in treatment modalities such as surgery, chemotherapy, radiotherapy and immunotherapy, its high recurrence rate and non‐responsiveness to treatment in advanced stages have not yet been effectively addressed [4]. Therefore, a deeper exploration of BC pathogenesis and effective therapeutic targets is urgently needed to improve the prognosis and clinical outcomes of patients with BC.

According to the Nomenclature Committee on Cell Death, cell death can be classified as accidental or regulated (RCD) [5]. Accidental cell death is caused by severe physical or chemical damage; it is passive and uncontrollable. In contrast, RCD is regulated by a series of signalling pathways that can be affected by genetic and pharmacological means. Therefore, RCD has received extensive attention in cancer research [6]. Ferroptosis is a novel RCD [7]; its morphological features, biochemical characteristics and regulatory pathways differ from those of other RCDs. Morphologically, ferroptotic cells exhibit mitochondrial shrinkage, reduction or absence of mitochondrial cristae; increased mitochondrial membrane density and rupture of the outer mitochondrial membrane, whereas the nucleus is barely altered [8]. Iron‐dependent lipid peroxidation is a biochemical feature that differentiates ferroptosis from other RCDs. Intracellularly enriched Fe^2+^ generates large amounts of reactive oxygen species (ROS) via the Fenton reaction, which catalyses the peroxidation of membrane lipids, ultimately destroying the normal structure of cell membranes and leading to cell death [9]. In addition, iron metabolism, lipid metabolism and antioxidant systems work in concert to coordinate the cellular sensitivity to ferroptosis [10]. Compared with normal cells, tumour cells require more iron for malignant proliferation, implying greater susceptibility to ferroptosis [11]. Recent studies have confirmed the important roles of ferroptosis in the development of cancers, such as BC, and that ferroptosis also contributes to the death of apoptosis‐resistant tumour cells [12]. The abnormal expression of ferroptosis‐related proteins in BC cells has good predictive value for the prognosis of BC. Moreover, chemotherapy drugs, traditional Chinese medicine and nano drugs might inhibit the progression of BC by inducing ferroptosis in BC cells [13]. Thus, ferroptosis is an important factor affecting BC progression and may be a potential therapeutic target for BC. However, the regulatory mechanisms underlying ferroptosis in BC remain largely unknown.

Discoidin domain receptor 1 (DDR1) is a transmembrane receptor tyrosine kinase (RTK). Similar to other RTKs, DDR1 contains an extracellular ligand‐binding domain, a short transmembrane domain and an intracellular kinase domain. However, unlike other RTKs, DDRs are the only collagen‐activated RTKs known to date and are thus also known as collagen receptors [14]. Unlike the rapid activation and inactivation of other RTKs, DDR1 exhibits slow kinase activation kinetics with sustained autophosphorylation [15]. DDR1 is predominantly expressed in epithelial cells and binds to virtually all known collagens, including collagen types I and IV, which are distributed in the interstitial matrix and basement membrane, respectively [16]. Abnormally high DDR1 expression has been observed in various cancers, including BC [17, 18]. DDR1 is known to be involved in the maintenance of proliferation, migration, invasion, stromal remodelling, apoptosis resistance, immune escape, and resistance to therapy [19] in tumour cells. Moreover, DDR1 is closely associated with the progression of hepatocellular carcinoma [20], lung cancer [21], and breast cancer [22]. However, the mechanism through which DDR1 regulates ferroptosis in BC remains unclear.

In this study, ferroptotic and ferroptosis‐resistant BC cells were screened, and the effects of DDR1 on BC ferroptosis and progression were investigated in vitro and in vivo. Homeobox A6 (HOXA6), an oncogenic protein [23, 24, 25, 26], was found to be upregulated in BC cells after ferroptosis using RNA sequencing (RNA‐seq). Experiments were conducted to determine whether DDR1 affects ferroptosis and BC progression by regulating HOXA6 expression.

## 2 Materials and Methods

### 2.1 Cell Culture and Treatment

Human BC cell lines RT4 (#iCell‐h184), 5637 (#iCell‐h232), SW780 (#iCell‐h278), J82COT (#iCell‐h115), T24 (#iCell‐h208) and TCCSUP (#iCell‐h384) were purchased from iCell Bioscience Inc. (Shanghai, China). All the cells were cultured in Dulbecco's‐modified Eagle medium (DMEM, #L120, BasalMedia, Shanghai, China) supplemented with 10% foetal bovine serum (FBS, #10099133C, Gibco, Carlsbad, USA) and 1% penicillin–streptomycin (#15070‐063, HyClone, Logan, USA), and were maintained in an incubator with 5% CO_2_ and a humidity of 70%–80% at 37°C.

To investigate the sensitivity of the different BC cell lines to the ferroptosis inducer erastin, the different BC cell lines were treated with different concentrations (0, 5, 10, 20 and 40 μM) of erastin (#S7242, Selleck, Huston, USA) and cultured for 48 h.

To further identify the primary type of cell death caused by erastin, the BC cells were treated with 10 μM ZVAD‐FMK (#HY‐16658B, an apoptosis inhibitor; MCE, Princeton, USA), 2 μM necrosulfonamide (#HY‐100573, a necrosis inhibitor; MCE) and 0.5 μM ferrostatin‐1 (#HY‐100579, a ferroptosis inhibitor; MCE) and then cultured for 48 h.

To knock down or over express DDR1 or HOXA6, small interfering RNAs (siRNAs) or overexpressing plasmids were transfected into TCCSUP or T24 cells using the Lipofectamine 2000 reagent (#11668019, Thermo, Waltham, USA) according to the manufacturer's instructions. The si‐negative control (si‐NC, sequencing: AACTCAGATCAAGCTCTGGTG), si‐DDR1 (sequencing: TACGTTTCTGTGGAGTAAATA) and si‐HOXA6 (sequencing: GACGTACACCTCACCTTGTTT) were synthesised and provided by Shanghai GenePharma Co. Ltd. (Shanghai, China). Empty plasmids (oe‐NC) and oe‐DDR1 were purchased from BioSune (Shanghai, China).

### 2.2 RNA Isolation, RNA‐Seq and Reverse Transcription Quantitative PCR (RT‐qPCR)

Total RNA of TCCSUP and T24 cells with different treatments was extracted using Trizol reagent (#15596018, Thermo, Waltham, USA), followed by reverse transcription of RNA to cDNA using the PrimeScript RT reagent Kit (#RR047Q, Takara, Kusatsu, Japan) with gDNA Eraser. Quantitative PCR was then performed using SYBR Premix Ex Taq (#RR420Q, Takara, Kusatsu, Japan). The relative mRNA levels were normalised against GAPDH, and the relative fold change was analysed by the 2^−∆∆Ct^ method. The sequences of all primers are listed in Table 1.

**TABLE 1:** Sequences of all primers.

Primer	Sequences (5′‐3′)
GAPDH	F: GTCTCCTCTGACTTCAACAGCG
	R: ACCACCCTGTTGCTGTAGCCAA
ACSL4	F: GCTATCTCCTCAGACACACCGA
	R: AGGTGCTCCAACTCTGCCAGTA
GPX4	F: ACAAGAACGGCTGCGTGGTGAA
	R: GCCACACACTTGTGGAGCTAGA
SLC7A11	F: TCCTGCTTTGGCTCCATGAACG
	R: AGAGGAGTGTGCTTGCGGACAT
DDR1	F: GATCTCGACTCCGCTTCAAGGA
	R: CAAAGGGTGTCCCTTACGCACA
HOXA6	F: AAAGCACTCCATGACGAAGGCG
	R: TCCTTCTCCAGCTCCAGTGTCT

RNA‐seq and analysis were performed by Shanghai Majorbio Bio‐Pharm Technology Co. Ltd. (Shanghai, China). Briefly, RNA samples were obtained from TCCSUP cells and TCCSUP cells treated with erastin. The KAPA Stranded RNA‐Seq Kit (Illumina, San Diego, CA, USA) was used to construct RNA‐seq libraries using multiplexing primers. An Illumina Nova sequencer (Illumina, San Diego, CA, USA) was used for RNA‐seq. Differential gene analyses were performed using the DESeq R package (ver. 1.10.1) with the raw gene count output from Rsubread. ∣Fold change∣ ≥ 2 and *p* < 0.05 were defined as differential genes.

### 2.3 Western Blot

Cells with different treatments were lysed in RIPA lysis buffer, and total protein concentrations were detected using a BCA assay kit (#23227, Pierce, Rockford, USA). Then, 20 μg protein samples were separated using 10% sodium dodecyl sulphate–polyacrylamide gel electrophoresis (SDS‐PAGE) and then electro‐transferred onto polyvinylidene fluoride (PVDF) membranes. The membranes were blocked with 5% skim milk at 37°C for 1 h and then incubated using primary antibodies against DDR1 (#SC‐374618, 1:1000, Santa Cruz Biotechnology, Dallas, USA), solute carrier family 7 member 11 (SLC7A11, #Ab175186, 1:1000, Abcam, Cambridge, UK), yes‐associated protein (YAP, #13584‐1‐AP, 1:2000, Proteintech, Rosemont, USA), p‐YAP (#29018‐1‐AP, 1:2000, Proteintech), acyl‐CoA synthetase long chain family member 4 (ACSL4, #Ab155282, 1:10000, Abcam), zinc finger E‐box binding homeobox 1 (ZEB1, #Sc‐515797, 1:1000, Santa Cruz Biotechnology), E‐cadherin (#20874‐1‐AP, 1:20000, Proteintech), glutathione peroxidase 4 (GPX4, #Ab125066, 1:1000, Abcam), neurofibromin 2 (NF2, #21686‐1‐AP, 1:2000, Proteintech), p‐NF2 (#28851‐1‐AP, 1:1000, Proteintech), HOXA6 (#18210‐1‐AP, 1:1000, Proteintech) and β‐actin (#66009–1‐Ig, 1:20000, Proteintech) overnight at 4°C. The following day, the blots were incubated with horseradish peroxidase (HRP)‐labelled secondary antibodies (#ab6728, #ab6721, #ab6741, 1:5000, Abcam) and visualised using an LAS‐4000 protein developing instrument (GE Healthcare, Pittsburgh, USA). Finally, Image J software (Media Cybernetics Inc.) was employed to scan the bands to obtain the grayscale values, and the protein expression was calculated as the grayscale of the target strip/the grayscale of the internal reference strip (β‐actin).

### 2.4 Immunofluorescence

Slides of TCCSUP and T24 cells subjected to different treatments were collected, washed with PBS and fixed with 4% paraformaldehyde. After permeabilisation by 0.5% Triton X‐100 for 20 min and blocking with normal goat serum at room temperature for 30 min, the cells were incubated with the primary antibody against DDR1 (#SC‐374618, Santa Cruz Biotechnology) at 4°C overnight. The cells were then rinsed with PBS and incubated with the fluorescent secondary antibody (donkey anti‐mouse IgG [H + L] highly cross‐adsorbed secondary antibody, Alexa Fluor 488, cat. no. A‐21202; Thermo Fisher Scientific) at room temperature in the dark for 1 h. After washing, DAPI was added, and the cells were incubated in the dark for 5 min. The cells were then sealed with a sealing solution containing an anti‐fluorescence quencher, and fluorescence images of TCCSUP and T24 cells were captured using a fluorescence microscope. Thereafter, three fields of view in the fluorescent sections of each group were randomly selected for image acquisition at 200× magnification. Finally, ImageJ was used to segment the green channel of the image and convert the fluorescence image into 8‐bit black and white images. The protein expression region was selected based on the threshold value, and the average grey value was calculated to represent the average fluorescence intensity [27].

### 2.5 Cell Counting Kit‐8 (CCK‐8) Assay

The BC cells were inoculated into a 96‐well plate at a density of 1 × 10^4^ cells/well, and after different treatments, each culture well was added to 10 μL of CCK‐8 solution (#40203ES60, Yeasen, Shanghai, China). After being cultured at 37°C for 4 h, the BC cells were placed in a microplate reader and the absorbance was measured at 450 nm.

### 2.6 Determination of Glutathione (GSH), Malondialdehyde (MDA) and Fe^2+^ Levels

Cells that were subjected to different treatments or tissue samples from the various groups were collected to assess their GSH, MDA and Fe^2+^ levels using the corresponding commercial GSH Content Assay Kit (#BC1175, Solarbio, Beijing, China), commercial MDA Content Assay Kit (#BC0025, Solarbio) and commercial Iron Content Assay Kit (#BC4355, Solarbio), respectively, in accordance with the manufacturer's instructions.

### 2.7 Mouse Xenograft Model

Male BALB/c nude mice were purchased from Shanghai Slack Experimental Animal Co. Ltd. (Shanghai, China). All the mice were housed in a laboratory animal room with a 12‐h light/12‐h dark cycle at 22°C ± 2°C and provided with adequate food and sterile water. After 1 week of acclimatisation, the mice were randomly divided into eight groups (*n* = 6): two control groups (TCCSUP and T24 xenograft), two erastin groups (TCCSUP and T24 xenograft) and one oe‐DDR1 + erastin, oe‐DDR1 + si‐HOXA6 + erastin, si‐DDR1 + erastin and si‐DDR1 + oe‐HOXA6 + erastin groups each. Briefly, 0.2 mL of a cell suspension containing 5 × 10^6^ TCCSUP or T24 cells was subcutaneously injected into the upper middle part of the groin. When the tumours reached 50 mm^3^, except for the mice in the control group, the mice in the other groups were intraperitoneally injected with erastin (40 mg/kg) twice daily until the end of the experiment. Tumour volumes were measured every 4 days. Mice were sacrificed on the 16th day after the first injection of erastin, and tumour weights were recorded. Xenograft tumours were collected for imaging, sizing, weighing and further analysis. All animal experimental procedures were approved by the Animal Experimental Ethics Committee of Ruijin Hospital, Shanghai Jiao Tong University School of Medicine.

### 2.8 Immunohistochemistry (IHC)

Tissue sections from the xenograft tumours were sequentially deparaffinised, hydrated and inactivated using endogenous peroxidase. Then, the sections were blocked with goat serum at room temperature for 10 min, and then incubated with the primary antibodies against GPX4 (#Ab125066, Abcam), SLC7A11 (#Ab175186, Abcam), ACSL4 (#Ab155282, Abcam), ZEB1 (#Sc‐515,797, Santa Cruz Biotechnology), E‐cadherin (#20874‐1‐AP, Proteintech), p‐YAP (#29018‐1‐AP, Proteintech) and p‐NF2 (#28851‐1‐AP, Proteintech) overnight at 4°C. The sections were then incubated with the secondary antibody, followed by dropwise addition of streptavidin–biotin peroxidase complex working solution. After staining with DAB chromogenic solution and haematoxylin, the sections were dehydrated and hyalinised, sealed with neutral gum and observed under a microscope. ImageJ was used to quantify the images [28].

### 2.9 Statistical Analysis

Each experiment was repeated thrice; the data are shown as mean ± SD. Statistical analysis and graphical plotting were performed using GraphPad Prism 7. Differences between both groups were analysed using an unpaired Student's *t*‐test. Differences between multiple groups were analysed using one‐way analysis of variance (ANOVA), followed by Tukey's post hoc test. *p* < 0.05 was considered statistically significant.

## 3 Results

### 3.1 Screening of Ferroptosis‐Sensitive and Ferroptosis‐Resistant BC Cells

To screen for ferroptosis‐sensitive and ferroptosis‐resistant BC cells, the BC cell lines 5637, SW780, TCCSUP, J82COT, T24 and RT4 cells were treated with different concentrations (0, 5, 10, 20, 40 μΜ) of erastin and cell viability was determined. As shown in Table 2, the viabilities of 5637, SW780, TCCSUP and RT4 cells decreased to different extents with increasing concentrations of erastin, whereas those of J82COT and T24 cells were not significantly affected (*p* > 0.05). The viability of TCCSUP cells decreased the most after erastin stimulation, whereas that of T24 cells remained almost unchanged. Therefore, TCCSUP and T24 cells were chosen as ferroptosis‐sensitive and ferroptosis‐resistant cells, respectively, for the subsequent experiments.

**TABLE 2:** The cell viability (OD values) of different bladder cancer cell lines treated with different concentrations of iron death inducer erastin.

Cell lines	Erastin dosage				
	0 μM	5 μM	10 μM	20 μM	40 μM
5637 cells (abs/AU)	3.44 ± 0.034	2.92 ± 0.029	2.59 ± 0.037	1.78 ± 0.017	1.59 ± 0.018
SW780 cells (abs/AU)	3.75 ± 0.029	3.74 ± 0.011	3.69 ± 0.006	3.80 ± 0.031	1.95 ± 0.042
TCCSUP cells (abs/AU)	2.77 ± 0.161	2.48 ± 0.098	2.05 ± 0.006	1.25 ± 0.007	0.87 ± 0.041
J82 cells (abs/AU)	3.45 ± 0.028	3.87 ± 0.022	4.07 ± 0.010	3.72 ± 0.017	2.70 ± 0.008
T24 cells (abs/AU)	3.77 ± 0.183	3.88 ± 0.019	4.14 ± 0.031	3.70 ± 0.228	3.86 ± 0.025
RT4 cells (abs/AU)	0.74 ± 0.006	0.76 ± 0.025	0.71 ± 0.020	0.54 ± 0.005	0.36 ± 0.009

### 3.2 DDR1 Expression in the Erastin‐Induced Ferroptosis‐Sensitive/Resistant Cells

**FIGURE 1:**
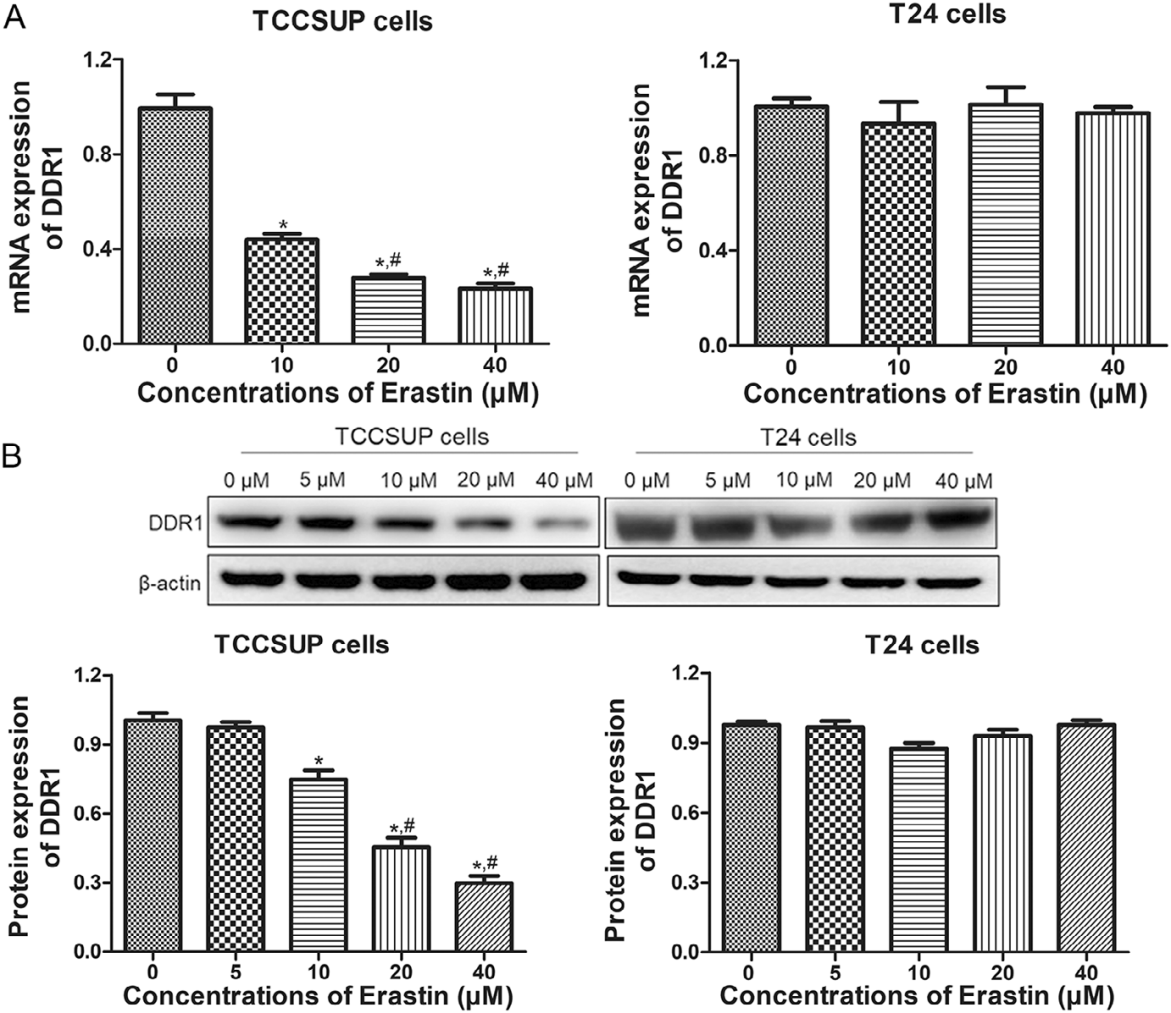
Erastin inhibits discoidin domain receptor 1 (DDR1) expression in ferroptosis‐sensitive bladder cancer (BC) cells, while it shows no effect on ferroptosis‐resistant BC cells. (A) *DDR1* mRNA expression in TCCSUP and T24 cells with different concentrations of erastin treatment, assessed using RT‐qPCR. (B) DDR1 protein levels in TCCSUP and T24 cells after erastin stimulation, assessed using western blotting. *N* = 3. *p < 0.05 versus 0 μM group, ^#^ *p* < 0.05 versus 10 μM group.

The expression of DDR1 in ferroptosis‐sensitive TCCSUP cells and ferroptosis‐resistant T24 cells was determined using RT‐qPCR and western blotting. The mRNA level of *DDR1* in TCCSUP cells gradually decreased as the erastin concentration increased (*p* < 0.05), whereas it remained unchanged in T24 cells (*p* > 0.05, Figure 1A). Subsequently, the protein expression of DDR1 was also assessed. Similarly, erastin downregulated DDR1 protein expression in a concentration‐dependent manner in TCCSUP cells (*p* < 0.05) but not in T24 cells (*p* > 0.05, Figure 1B). These results suggest that erastin downregulates DDR1 expression in ferroptosis‐sensitive BC cells and that DDR1 may play an important role in erastin‐induced ferroptosis in BC cells.

### 3.3 Effects of DDR1 on the Erastin‐Induced Ferroptosis in BC Cells

**FIGURE 2:**
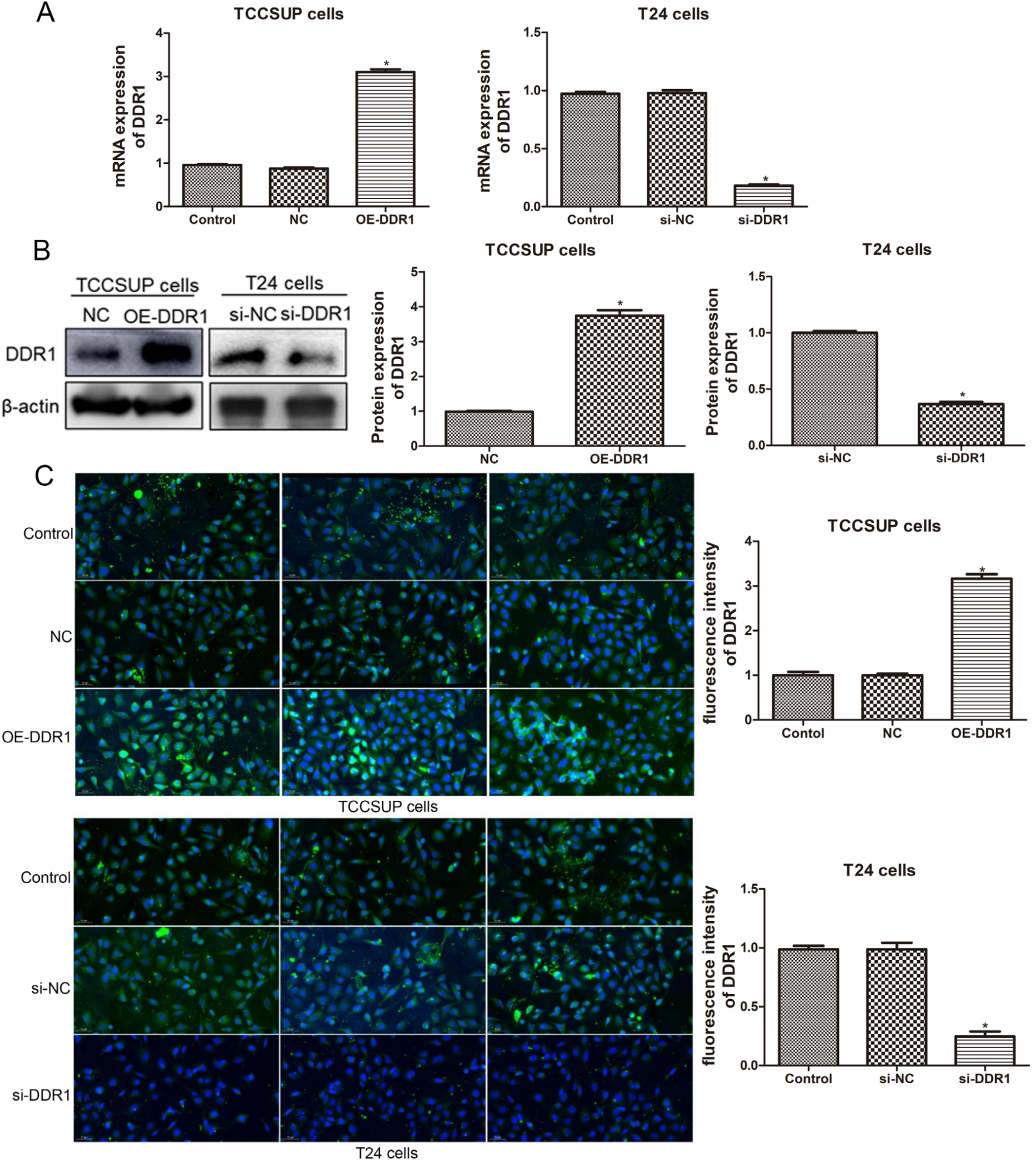
Cell transfection efficiency after transfection with si‐DDR1 and oe‐DDR1 in TCCSUP cells and T24 cells. (A) RT‐qPCR analysis of *DDR1* mRNA expression in TCCSUP cells after DDR1 overexpression and T24 cells upon DDR1 knockdown. (B) Western blot (WB) analysis and quantification of DDR1 protein level in TCCSUP and T24 cells. (C) Representative immunofluorescence (IF) staining and quantification of DDR1 in TCCSUP and T24 cells. *N* = 3. *p < 0.05 versus control group.

To clarify the role of DDR1 in the regulation of ferroptosis in different BC cells, TCCSUP cells with DDR1 overexpression and T24 cells with DDR1 knockdown were constructed. No significant differences were noted in DDR1 expression between the control and NC/si‐NC groups of TCCSUP and T24 cells (*p* > 0.05, Figure 2A). Compared with the control group, the mRNA and protein expression of DDR1 were both significantly upregulated in the oe‐DDR1 group of TCCSUP cells (*p* < 0.05), whereas they were downregulated in the si‐DDR1 group of T24 cells (*p* < 0.05, Figure 2A,B). Additionally, the trend of DDR1 fluorescence intensity in different TCCSUP or T24 cells was similar to that of *DDR1* mRNA expression in different cells (Figure 2C). These results indicate that TCCSUP cells overexpressing DDR1 and T24 cells with DDR1 knockdown have been successfully established.

The effect of DDR1 on the viability of erastin‐treated BC cells was measured using the CCK‐8 assay. DDR1 overexpression significantly increased the viability of erastin‐treated TCCSUP cells, whereas DDR1 knockdown had the opposite effect on erastin‐treated T24 cells (Table 3). These results indicate that DDR1 overexpression decreased the sensitivity of TCCSUP cells to erastin, whereas DDR1 knockdown enhanced the sensitivity of T24 cells to erastin.

**TABLE 3:** The cell viability (OD values) of erastin‐induced bladder cancer cells after being transfected with si‐DDR1 or OE‐DDR1.

Cell lines	Erastin dosage				
	0 μM	5 μM	10 μM	20 μM	40 μM
TCCSUP cells					
Control (abs/AU)	2.92 ± 0.056	2.79 ± 0.078	2.33 ± 0.037	1.68 ± 0.029	1.27 ± 0.092
NC (abs/AU)	2.90 ± 0.092	2.72 ± 0.088	2.24 ± 0.091	1.55 ± 0.045	1.18 ± 0.055
OE‐DDR1 (abs/AU)	3.30 ± 0.054	3.13 ± 0.065	2.90 ± 0.027	2.62 ± 0.029	2.49 ± 0.042
T24 cells					
Control (abs/AU)	3.19 ± 0.069	2.90 ± 0.028	2.71 ± 0.067	2.38 ± 0.106	2.36 ± 0.041
si‐NC (abs/AU)	3.27 ± 0.042	3.00 ± 0.017	3.09 ± 0.016	2.67 ± 0.121	2.54 ± 0.032
si‐DDR1 (abs/AU)	2.84 ± 0.072	2.55 ± 0.040	2.25 ± 0.051	1.50 ± 0.060	1.06 ± 0.059

**FIGURE 3:**
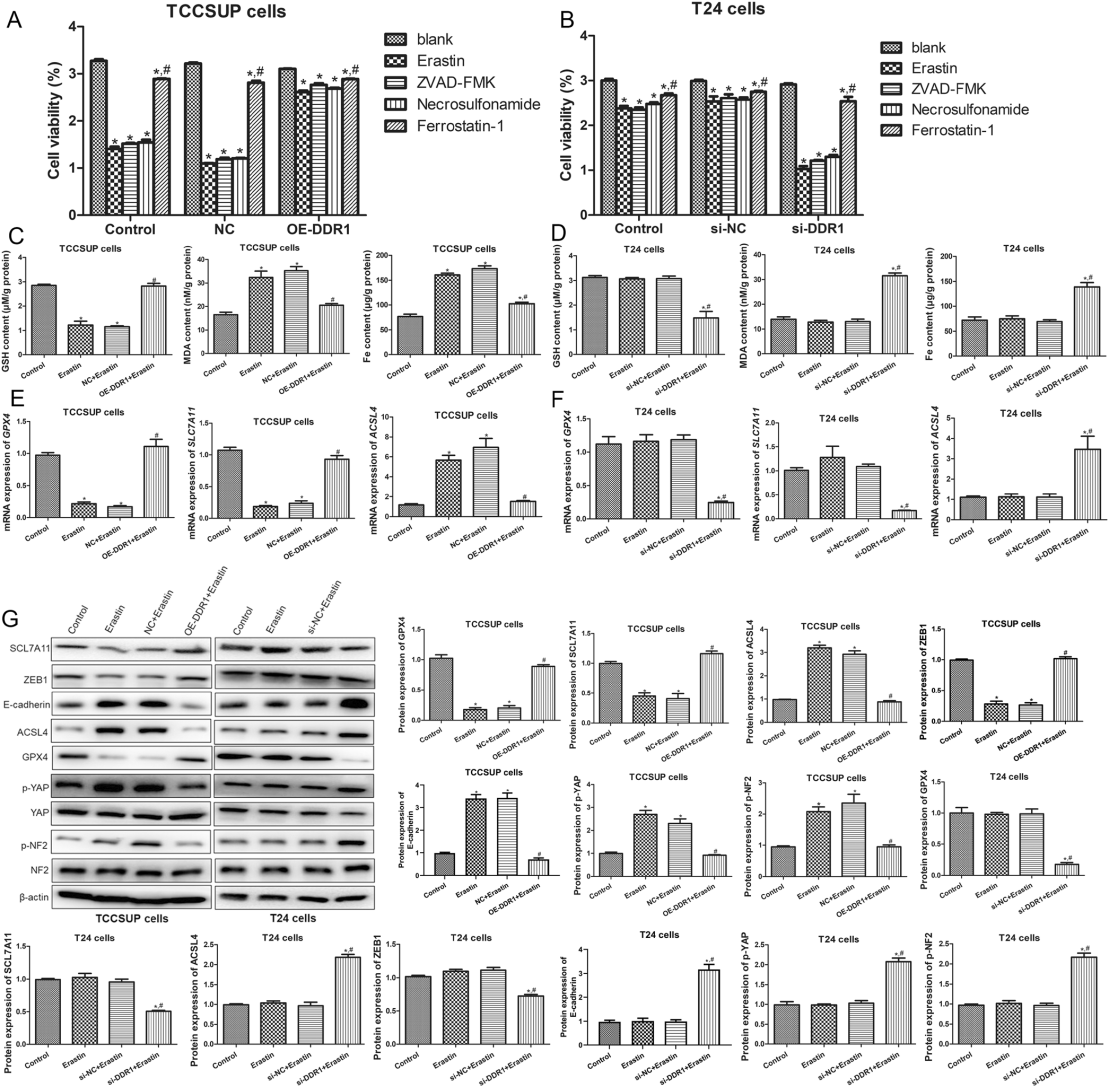
DDR1 inhibits ferroptosis of BC cells. (A) CCK‐8 assay of TCCSUP cells treated with erastin, apoptosis inhibitor ZVAD‐FMK, necroptosis inhibitor necrosulfonamide or ferroptosis inhibitor ferrostatin‐1 combined with NC and OE‐DDR1. (B) CCK‐8 assay of T24 cells treated with erastin, apoptosis inhibitor ZVAD‐FMK, necroptosis inhibitor necrosulfonamide or ferroptosis inhibitor ferrostatin‐1 combined with NC and OE‐DDR1. (C) Levels of glutathione (GSH), malondialdehyde (MDA) and Fe^2+^ in TCCSUP cells. (D) Levels of GSH, MDA and Fe^2+^ in T24 cells. (E) RT‐qPCR analysis of glutathione peroxidase 4 (*GPX4*), solute carrier family 7 member 11 (*SLC7A11*) and acyl‐CoA synthetase long chain family member 4 (*ACSL4*) expression in TCCSUP cells. (F) RT‐qPCR analysis of *GPX4*, *SLC7A11* and *ACSL4* expression in T24 cells. (G) WB analysis and quantification of SLC7A11, zinc finger E‐box binding homeobox 1 (ZEB1), E‐cadherin, ACSL4, GPX4, yes‐associated protein (YAP), p‐YAP, neurofibromin 2 (NF2), and p‐NF2 expression in TCCSUP and T24 cells. *N* = 3. *p < 0.05 versus control group. ^#^ *p* < 0.05 versus NC + erastin or si‐NC + erastin group.

To confirm the main types of cell death induced by erastin, ZVAD‐FMK, necrosulfonamide and ferrostatin‐1 were used. In TCCSUP cells, the apoptosis inhibitors ZVAD‐FMK and necrosulfonamide had no significant effects on the viability of erastin‐treated cells (*p* > 0.05), whereas the ferroptosis inhibitor ferrostatin‐1 reversed the cell viability loss caused by erastin (*p* < 0.05, Figure 3A). This phenomenon was also observed in T24 cells; the knockdown of DDR1 expression with an iron death inhibitor significantly improved cell viability (Figure 3B). This implies that the primary mode of cell death induced by erastin is ferroptosis.

Ferroptosis‐related mechanisms in TCCSUP and T24 cells were determined. Compared to the control cells, the level of GSH was decreased and that of MDA and Fe^2+^ notably increased in TCCSUP cells after erastin treatment (*p* < 0.05), whereas DDR1 overexpression restored GSH levels to a level similar to that of the control cells (*p* > 0.05, Figure 3C). In T24 cells, erastin treatment did not significantly affect the levels of GSH, MDA and Fe^2+^ compared to those in control T24 cells (*p* > 0.05), whereas DDR1 knockdown decreased GSH levels and increased MDA and Fe^2+^ levels (*p* < 0.05, Figure 3D). The expression levels of GPX4, SCL7A11, ACSL4, epithelial–mesenchymal transition (EMT)–related proteins (ZEB1 and E‐cadherin) and NF2‐YAP signalling pathway proteins (p‐YAP and p‐NF2) in TCCSUP and T24 cells were analysed. In comparison with control TCCSUP cells, erastin induction significantly downregulated the expression of GPX4, SLC7A11 and ZEB1 (*p* < 0.05) and upregulated that of ACSL4, E‐cadherin, p‐YAP and p‐NF2 (*p* < 0.05, Figure 3E,G). However, DDR1 overexpression in TCCSUP cells reversed the erastin‐induced changes in expression levels (Figure 3E,G). In T24 cells, no significant differences were found in the expression of all related genes and proteins among the control, erastin and si‐DDR1 + erastin groups (*p* > 0.05; Figure 3F,G). DDR1 silencing in T24 cells markedly downregulated GPX4, SLC7A11 and ZEB1 and upregulated ACSL4, E‐cadherin, p‐YAP and p‐NF2 (*p* < 0.05, Figure 3F,G). These results suggested that DDR1 overexpression inhibited ferroptosis in ferroptosis‐sensitive BC cells.

### 3.4 Sequencing Analysis and the Interaction Between DDR1 and HOXA6

**FIGURE 4:**
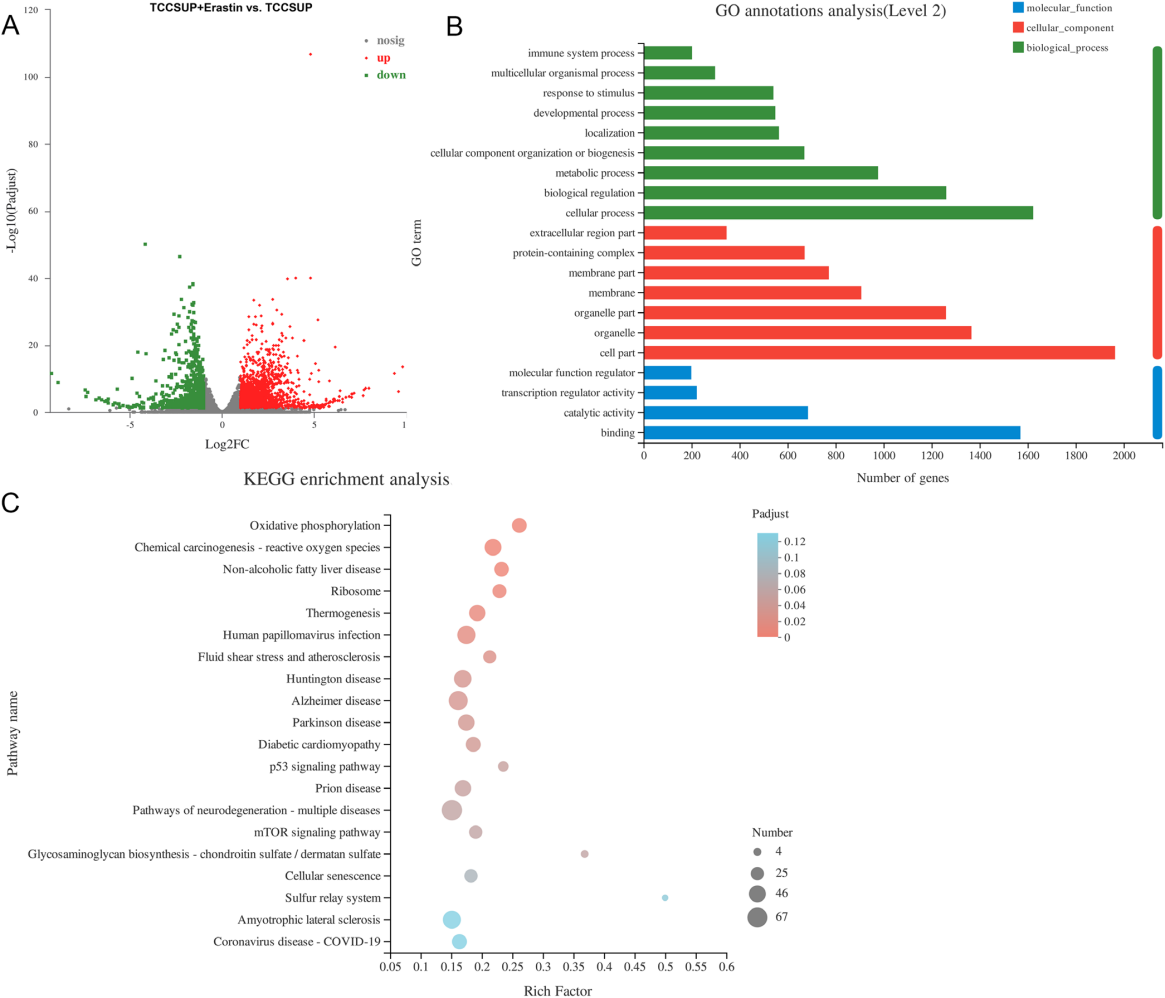
Identification and functional analysis of differentially expressed genes (DEGs) in TCCSUP cells and erastin‐treated TCCSUP cells (*N* = 3). (A) Volcano plot of DEGs detected by RNA‐sequencing (RNA‐seq) between TCCSUP cells and erastin‐treated TCCSUP cells. Red and green dots represent ∣fold change∣ ≥ 2 and *p* < 0.05. (B) Gene Ontology (GO) annotation analysis of DEGs between TCCSUP cells and erastin‐treated TCCSUP cells. (C) Kyoto Encyclopedia of Genes and Genomes (KEGG) enrichment analysis of DEGs between TCCSUP cells and erastin‐treated TCCSUP cells.

To investigate the mechanisms by which DDR1 regulates ferroptosis in ferroptosis‐sensitive BC cells, TCCSUP and erastin‐treated TCCSUP cells were collected for RNA‐seq, and 2558 differentially expressed genes (DEGs) were identified, with 1831 upregulated and 727 downregulated genes (Figure 4A). In particular, HOXA6 was significantly increased after erastin treatment (Figure 4A), and DDR1 was downregulated in erastin‐treated TCCSUP cells compared to that in untreated TCCSUP cells (Figure S1A). Gene Ontology (GO) annotation analysis showed that the DEGs were enriched in ‘cellular processes’, ‘biological regulation’, ‘metabolic processes’ and other biological processes; ‘cell part’, ‘organelle’, ‘organelle part’ and other cellular components; and ‘binding’, ‘catalytic activity’, ‘transcription regulator activity’ and other molecular functions (Figure 4B). Kyoto Encyclopedia of Genes and Genomes (KEGG) enrichment analysis showed that DEGs were enriched in ‘oxidative phosphorylation’, ‘chemical carcinogenesis‐ROS’, ‘non‐alcoholic fatty liver disease’, ‘p53 signalling pathway’ and ‘mTOR signalling pathway’. (Figure 4C).

**FIGURE 5:**
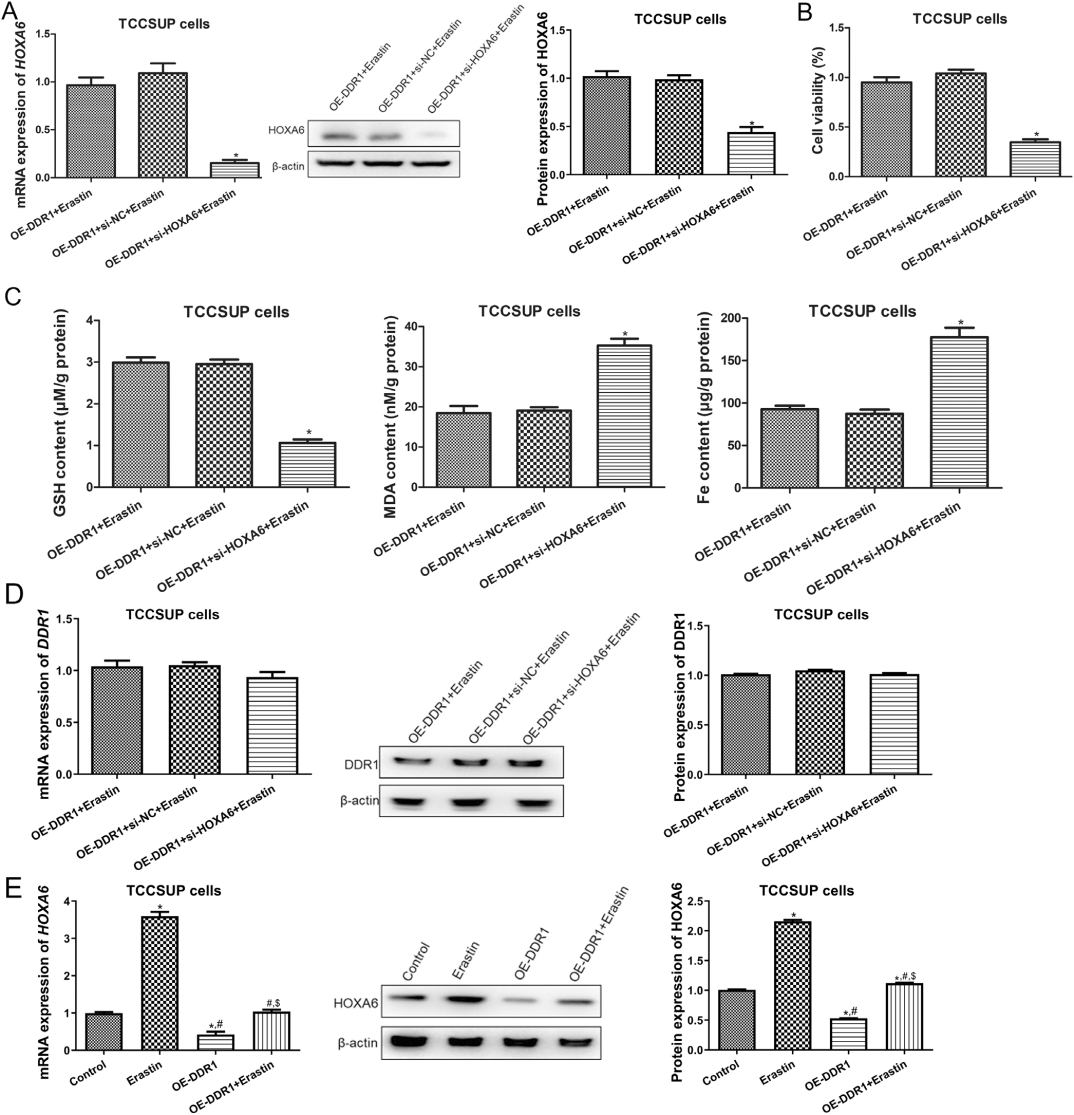
DDR1 inhibits ferroptosis of BC cells by regulating homeobox A6 (HOXA6). (A) Left: RT‐qPCR analysis of *HOXA6* mRNA expression in TCCSUP cells with or without HOXA6 silencing. Right: WB analysis and quantification of HOXA6 protein level in TCCSUP cells. (B) CCK‐8 assay of TCCSUP cells. (C) Levels of GSH, MDA and Fe^2+^ in TCCSUP cells. (D) The expression of DDR1 in TCCSUP cells with or without HOXA6 silencing using RT‐qPCR and western blot. *N* = 3. *p < 0.05 versus OE‐DDR1 + erastin group. (E) RT‐qPCR and western blot used to measure the expression of HOXA6 in TCCSUP cells with erastion and DDR1 overexpresssion. *N* = 3. *p < 0.05 versus control. ^#^ *p* < 0.05 versus erastin. ^$^ *p* < 0.05 versus OE‐DDR1.

HOXA6 expression is dysregulated in multiple cancers, including renal cancer [25], colorectal cancer [24] and glioma [29]. In addition, HOXA6 regulates the proliferation, migration and invasion of cancer cells [24, 26]; however, its role in ferroptosis remains unknown. Western blotting results showed that, compared with TCCSUP cells, HOXA6 was significantly upregulated in erastin‐treated TCCSUP cells (*p* < 0.05, Figure S1B), and overexpression of DDR1 significantly downregulated HOXA6 expression (*p* < 0.05, Figure S1C). To elucidate whether DDR1 affects ferroptosis in BC cells by regulating HOXA6, we knocked down HOXA6 in TCCSUP cells overexpressing DDR1. HOXA6 mRNA and protein levels were significantly decreased in the oe‐DDR1 + si‐HOXA6 + erastin group compared with those in the oe‐DDR1 + erastin group (*p* < 0.05, Figure 5A). Upon HOXA6 knockdown, the loss of cell viability induced by OE‐DDR1 and erastin was ameliorated (*p* < 0.05, Figure 5B), accompanied by downregulation of GSH and upregulation of MDA and Fe^2+^ (*p* < 0.05, Figure 5C). Additionally, no significant differences were found in DDR1 mRNA and protein expression among the OE‐DDR1 + erastin, DDR1 + si‐NC + erastin and DDR1 + si‐HOXA6 + erastin groups (*p* > 0.05, Figure 5D). Furthermore, erastin treatment significantly upregulated HOXA6 mRNA and protein expression compared to those in control TCCSUP cells (*p* < 0.05), whereas DDR1 overexpression downregulated erastin‐induced HOXA6 mRNA and protein expression in TCCSUP cells (*p* < 0.05, Figure 5E). These results indicate that HOXA6 may be a downstream target of DDR1 and that DDR1 may negatively regulate HOXA6 and inhibit ferroptosis in BC cells by targeting HOXA6.

### 3.5 DDR1 Facilitates BC Progression and Inhibits BC Ferroptosis Targeting HOXA6

**FIGURE 6:**
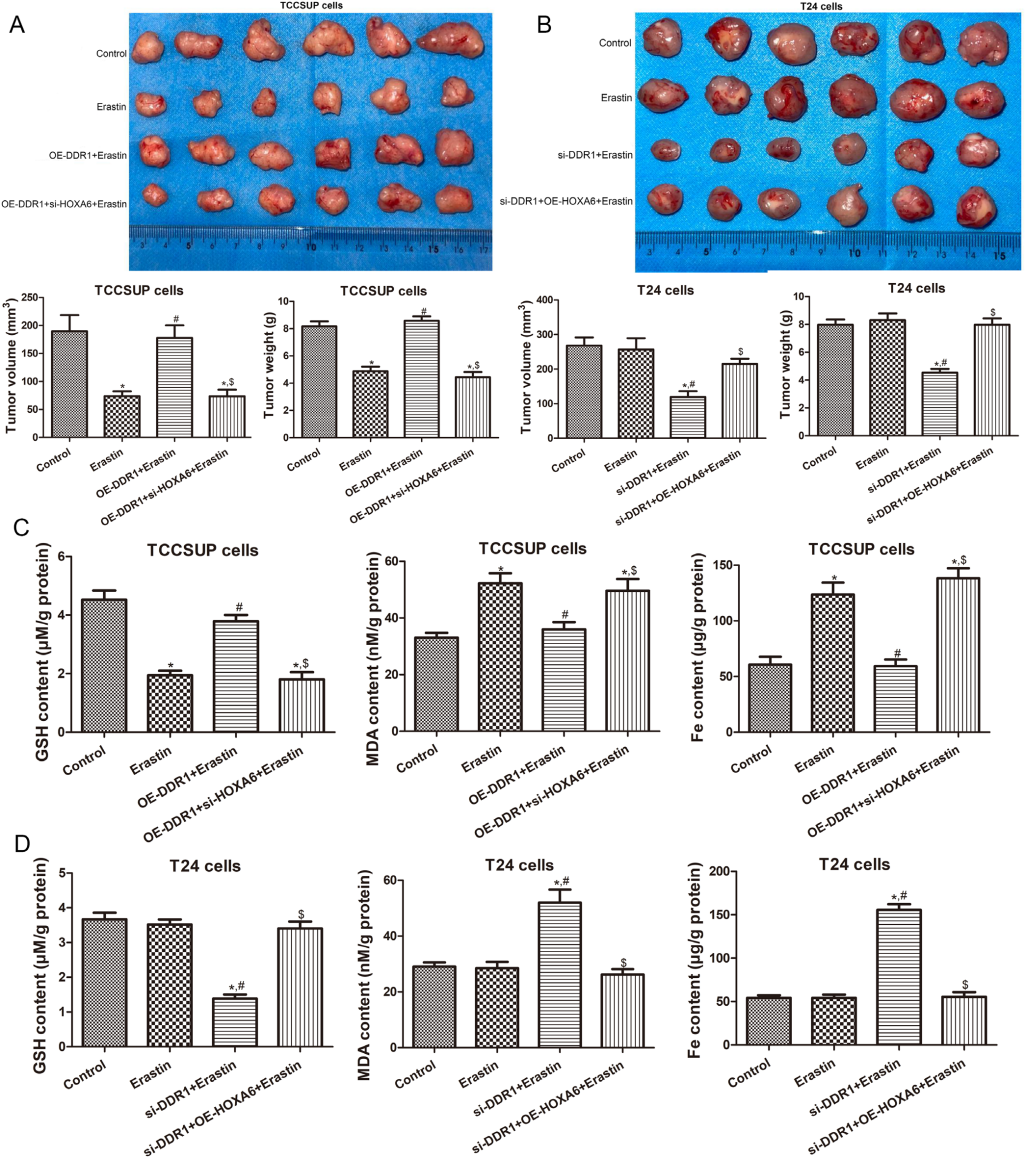
DDR1 targeting HOXA6 facilitates BC growth and inhibits BC ferroptosis in vivo. (A) Nude mice were xenografted with TCCSUP cells and treated with or without erastin. The subcutaneous xenografts (upper), tumour volumes (lower left) and tumour weights (lower right) are shown. (B) Nude mice were xenografted with T24 cells and treated with or without erastin. The subcutaneous xenografts (upper), tumour volumes (lower left) and tumour weights (lower right) are shown. (C) Levels of tumoural GSH, MDA and Fe^2+^ in the TCCSUP xenograft group. (D) Levels of tumoural GSH, MDA and Fe^2+^ in the T24 xenograft group. *N* = 3. *p < 0.05 versus control group. ^#^ *p* < 0.05 versus erastin group. ^$^ *p* < 0.05 versus OE‐DDR1 + erastin group.

To explore the regulatory role of DDR1 in ferroptosis and BC progression in vivo, a subcutaneous tumour‐bearing mouse model was constructed in nude mice. In the ferroptosis‐sensitive TCCSUP xenograft group, the ferroptosis inducer erastin significantly reduced the volume and weight of the xenograft tumours compared with those in control mice (*p* < 0.05), whereas DDR1 overexpression evidently increased the tumour size and weight caused by erastin (*p* < 0.05), and the tumour size and weight were reduced by HOXA6 knockdown (Figure 6A). In the ferroptosis‐resistant T24 cell xenograft group, erastin treatment did not significantly influence tumour size and weight compared to the control mice (*p* > 0.05). However, after DDR1 knockdown, tumour growth was inhibited, and HOXA6 overexpression reversed the action of DDR1 silencing (Figure 6B).

**FIGURE 7:**
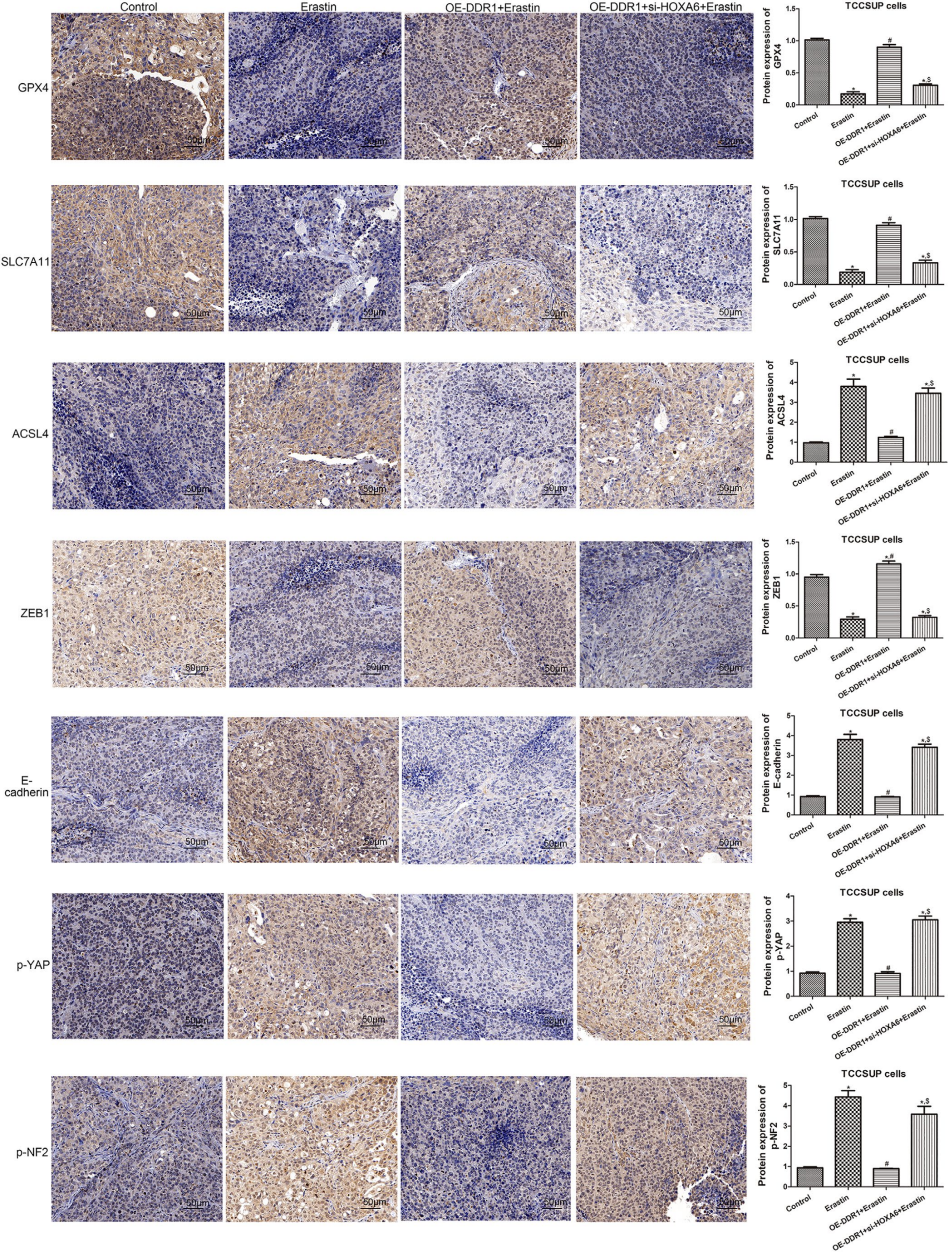
Effect of DDR1 on the levels of proteins involved in ferroptosis, EMT and NF2‐YAP pathway in TCCSUP xenografts. Representative immunohistochemistry (IHC) staining and quantification of GPX4, SLC7A11, ACSL4, ZEB1, E‐cadherin, p‐YAP and p‐NF2 in tumours of the TCCSUP xenograft group. *N* = 3. *p < 0.05 versus control group. *p < 0.05 versus control group. ^#^ *p* < 0.05 versus erastin group. ^$^ *p* < 0.05 versus OE‐DDR1 + erastin group.

**FIGURE 8:**
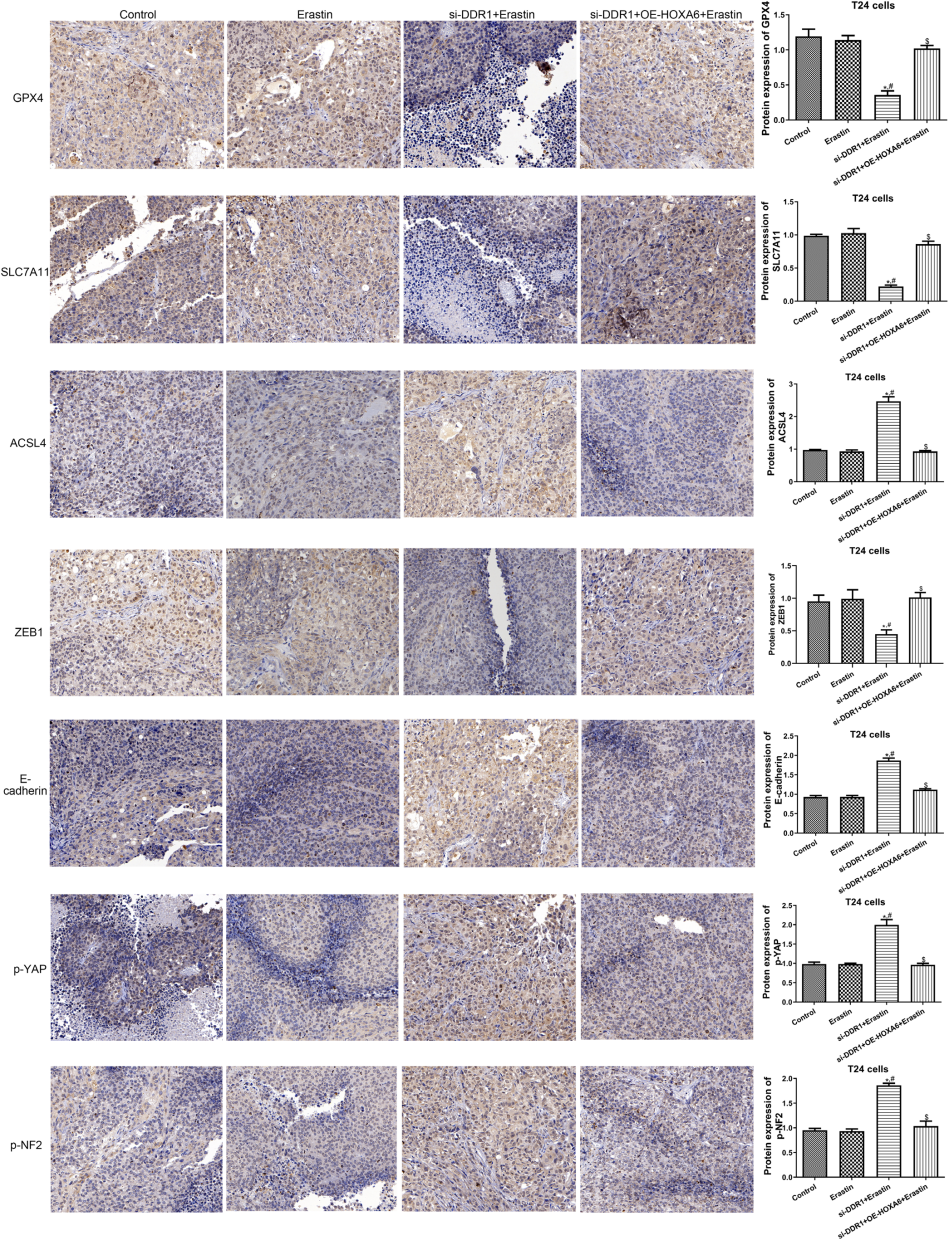
Effect of DDR1 on the levels of proteins involved in ferroptosis, EMT and NF2–YAP pathway in T24 xenografts. Representative IHC staining and quantification of GPX4, SLC7A11, ACSL4, ZEB1, E‐cadherin, p‐YAP and p‐NF2 in T24 xenograft group tumours. *N* = 3. *p < 0.05 versus control group. *p < 0.05 versus control group. ^#^ *p* < 0.05 versus erastin group. ^$^ *p* < 0.05 versus OE‐DDR1 + erastin group.

Furthermore, the underlying mechanisms were investigated. In mice treated with TCCSUP cells, erastin significantly decreased GSH levels, as well as GPX4, SLC7A11 and ZEB1 expression, and increased MDA and Fe^2+^ levels, as well as ACSL4, E‐cadherin, p‐YAP and p‐NF2 expression (Figure 6C, Figure 7). These effects were ablated by DDR1 overexpression, whereas HOXA6 knockdown reversed the effects of DDR1 overexpression (Figure 6C, Figure 7). Meanwhile, in mice treated with T24 cells, erastin had no effect on GSH, MDA and Fe^2+^ levels or the expression of GPX4, SLC7A11, ACSL4, ZEB1, E‐cadherin, p‐YAP and p‐NF2 (Figure 6D, Figure 8) in tumours. However, DDR1 knockdown significantly reduced GSH levels and GPX4, SLC7A11 and ZEB1 expression and increased MDA and Fe^2+^ levels, as well as ACSL4, E‐cadherin, p‐YAP and p‐NF2 expression levels in tumour tissues (Figure 6D, Figure 8). However, HOXA6 overexpression abolished the effects of DDR1 silencing (Figure 6D, Figure 8). These results imply that DDR1 overexpression promotes BC tumour growth by inhibiting ferroptosis and targeting HOXA6.

## 4 Discussion

BC is a life‐threatening disease with few effective treatment options. Recently, ferroptosis has been shown to play a crucial role in the progression of BC; however, the regulatory mechanisms of BC ferroptosis are largely unknown. In this study, ferroptosis‐sensitive TCCSUP and ferroptosis‐resistant T24 cells were screened. DDR1 expression decreased in TCCSUP cells in a concentration‐dependent manner after erastin treatment. DDR1 overexpression inhibited ferroptosis in TCCSUP cells, whereas DDR1 knockdown promoted ferroptosis in T24 cells. Mechanistically, DDR1 inhibits ferroptosis in BC cells by regulating HOXA6 expression, which ultimately promotes BC progression. Therefore, DDR1 is an important protein modulating BC ferroptosis in BC.

Ferroptosis is an iron‐dependent, lipid peroxidation‐induced RCD. Tumour cells are often abundant in iron because of their high metabolic activity. As such, inducing ferroptosis can promote the death of tumour cells, especially apoptosis‐ and autophagy‐resistant tumour cells, thus inhibiting tumour progression [30, 31]. Erastin is a well‐known ferroptosis inducer. In this study, various RCD inhibitors were used to treat BC cells after erastin stimulation. Only ferroptosis inhibitors abolished erastin‐induced cell death. In addition, we performed RNA‐seq on TCCSUP cells before and after erastin treatment and found that DEGs were significantly enriched in the p53 and mTOR signalling pathways. The tumour suppressor p53 plays a bidirectional regulatory role in ferroptosis through GPX4‐dependent or GPX4‐independent pathways [32]. In contrast, mTOR regulates ferroptosis through autophagy‐dependent or autophagy‐independent mechanisms [33]. Thus, the p53 and mTOR signalling pathways may play important roles in erastin‐induced ferroptosis. Ferroptosis is a key pathway hindering BC progression. Kong et al. found that baicalin‐induced BC cell death was accompanied by ROS and iron accumulation. Among various RCD inhibitors tested, only the ferroptosis inhibitor abrogated baicalin‐induced BC cell death [34]. The delivery of iron oxide nanoparticles can promote ferroptosis in BC cells by increasing their labile iron pool and ultimately inhibiting BC progression [35]. Xu et al. found that abietic acid promotes ferroptosis of BC cells by downregulating GPX4, whereas abietic acid synergizes with anti‐BC chemotherapeutic agents to suppress BC growth in vivo [36]. Thus, inducing ferroptosis is an important way to inhibit BC progression.

However, our results suggested that not all BC cells are sensitive to ferroptosis. The viability of 5637, SW780, TCCSUP and RT4 cells decreased as the erastin concentration increased, whereas that of J82COT and T24 cells was unaffected. BC cells achieve ferroptosis resistance through multiple mechanisms. MAF transcription factor G antisense RNA 1 (MAFG‐AS1) inhibited BC ferroptosis and promotes cisplatin resistance by stabilising poly(rC)‐binding protein 2 (PCBP2) and thus interacting with ferroportin 1 (FPN1) [37]. Phosphoglycerol dehydrogenase (PHGDH) enhances SLC7A11 expression by interacting with PCBP2, which ultimately inhibits ferroptosis and promotes malignant progression of BC [38]. Liu et al. confirmed that the loss of epithelial membrane protein 1 (EMP1), which leads to the upregulation of SLC7A11 expression, is important for inducing ferroptosis resistance in BC cells [39]. Although the studies described above explored the molecular mechanisms of BC cell ferroptosis resistance in BC cells, the regulatory pathways of BC cell ferroptosis remain largely unclear. Thus, an in‐depth exploration of the molecules that regulate ferroptosis in BC cells is essential for the development of novel therapeutic options for BC.

In this study, we identified DDR1 as an important protein affecting the sensitivity of BC cells to ferroptosis. Our results confirmed that DDR1 expression in ferroptosis‐sensitive BC cells gradually decreased with increasing erastin concentration, while that in ferroptosis‐resistant BC cells was not affected. As a collagen receptor, DDR1 plays a crucial cellular role in detecting collagen signalling in the surrounding environment, and its expression is dysregulated in various cancers. DDR1 is a prognostic marker for non‐small cell lung cancer (NSCLC), and its upregulation is significantly associated with lymph node metastasis of NSCLC [40]. DDR1 levels are also significantly upregulated in ovarian [41] and gastric [42] cancers. In BC, DDR1 expression is significantly increased; DDR1 overexpression promotes the proliferation, migration and invasion of BC cells [43, 44]. In addition, high DDR1 expression is associated with a poor prognosis in patients with BC [43]. However, the role of DDR1 in ferroptosis is currently unknown. Our study found that in ferroptosis‐sensitive BC cells, DDR1 expression gradually decreased with increasing concentrations of the ferroptosis inducer erastin, whereas DDR1 expression in ferroptosis‐resistant BC cells was unaffected by erastin, suggesting that DDR1 is associated with ferroptosis. Silencing DDR1 decreased BC cell survival, but a ferroptosis inhibitor abolished cell death caused by DDR1 downregulation, implying that DDR1‐induced cell survival is associated with ferroptosis resistance.

We also examined the expression of various ferroptosis‐related markers. GSH is a crucial intracellular antioxidant [45] and its synthesis is dependent on the intracellular transport of cysteine mediated by SLC7A11 [46]. GPX4, in contrast, catalyses the reduction of lipid peroxide by GSH to nontoxic lipid alcohols [47]. Hence, the antioxidant system centred on the SLC7A11/GSH/GPX4 axis is an important mechanism protecting cells from ferroptosis [48]. Fe^2+^ is a substrate of the Fenton reaction [49], whereas MDA is an important by‐product of lipid peroxidation [50]. ACSL4 is a rate‐limiting enzyme that catalyses the synthesis of phospholipids containing polyunsaturated fatty acids (PUFA‐PLs). PUFA‐PLs are the core components of membrane lipids and substrates of the lipid peroxidation reaction [51]. Therefore, Fe^2+^, MDA and ACSL4 are potential markers of enhanced ferroptosis. Our study revealed that DDR1 overexpression resulted in the upregulation of GSH, GPX4 and SLC7A11 expression and downregulation of MDA, Fe^2+^ and ACSL4 expression in BC cells. DDR1 knockdown exhibited the opposite effect. These results suggest that DDR1 overexpression inhibits BC cell ferroptosis, whereas DDR1 silencing promotes BC cell ferroptosis. Our results also revealed that DDR1 promoted EMT (downregulation of E‐cadherin and upregulation of ZEB1) and inhibited NF2–YAP signalling pathway activation in BC cells. Overlapping molecules and pathways have been shown to regulate EMT and ferroptosis [52], whereas the NF2–YAP pathway promotes ferroptosis by upregulating ACSL4 [53]. Xie et al. [44] and Azizi et al. [54] found that DDR1 activates Zeb1 expression. In BC and prostate cancer cell lines, DDR1 activated proliferation. Corroborating the Zeb1 inhibition of E‐cadherin expression, Azizi et al. [54] found that DDR1 induces E‐cadherin downregulation and N‐cadherin upregulation. Yeh et al. [55] found that DDR1 increased E‐cadherin expression but decreased the proliferation of renal cells. Depending on the cell type and tumour origin, DDR1 may be a pro‐ or anti‐tumour receptor; this warrants further investigation. Taken together, we hypothesised that DDR1 inhibits ferroptosis by regulating the EMT and NF2–YAP signalling pathways in BC cells. Collectively, our study confirmed that DDR1 is a key protein in the regulation of ferroptosis in BC cells.

To further clarify the mechanism by which DDR1 regulates ferroptosis in BC cells, we performed RNA‐seq on TCCSUP cells and *HOXA6*, a tumour‐associated gene. HOXA6 is a member of the HOX family [23] and its expression is dysregulated in colorectal cancer [24], renal cancer [25], glioma [29] and gastric cancer [26]. Some studies have shown that HOXA6 promotes the malignant phenotypes of tumour cells, such as proliferation, migration and invasion [24, 26], whereas others have confirmed that HOXA6 inhibits tumour cell proliferation and promotes apoptosis [25]. However, the role of HOXA6 in ferroptosis remains unclear. Our study found that the expression of HOXA6 in BC cells was significantly increased after erastin treatment; thus, we hypothesised that HOXA6 might be involved in ferroptosis regulation. DDR1‐induced ferroptosis resistance in BC cells was ablated after HOXA6 knockdown, suggesting that DDR1 inhibits ferroptosis in BC cells by regulating HOXA6. Additionally, we examined the effects of DDR1 and HOXA6 on BC ferroptosis in vivo. Erastin inhibits the growth of ferroptosis‐sensitive BC cells but not ferroptosis‐resistant BC cells in nude mice. Overexpression of DDR1 abolished the erastin‐induced inhibition of BC growth, while ferroptosis was significantly reduced in tumour tissues, which further confirmed the inhibitory effect of DDR1 on BC ferroptosis in vivo. Meanwhile, knockdown of HOXA6 abolished DDR1‐mediated BC growth and BC ferroptosis inhibition, indicating that DDR1 targets HOXA6 to regulate BC ferroptosis, and DDR1 promoted BC progression by regulating HOXA6. Dai et al. [56] reported that DDR1 expression was significantly higher in dry eye corneas than in normal controls and that DDR1‐IN‐1 (a DDR1 inhibitor) treatment could improve dry eye symptoms by decreasing lipid hydroperoxide levels and inhibiting the expression of ferroptosis markers, particularly ACSL4. Additionally, overexpression or reactivation of YAP diminishes the protective effects of DDR1‐IN‐1, suggesting that the Hippo/YAP pathway is involved in DDR1‐targeted therapeutic effects [56]. These findings contradict the results of this study. This may be because the cell lines are different and the cell line itself is ferroptotic (the cells used in our study were first treated with the ferroptosis inducer erastin). The specific reasons for this warrant further investigation.

This study has certain limitations. First, experiments on control cells OE‐DDR1 (for TCCSUP) or si‐DDR1 (for T24) +/− siHOXA6 in the absence of erastin need to be conducted in the future. Additionally, we did not explore how DDR1 regulates HOXA6 expression or the molecular mechanism by which HOXA6 regulates ferroptosis in BC cells. Whether other genes and pathways are involved in regulating ferroptosis following DDR1 overexpression or HOXA6 knockdown remains unclear. We also did not verify a correlation between DDR1, HOXA6 and ferroptosis in human BC samples. Further studies are required to explore these issues.

## 5 Conclusions

**FIGURE 9:**
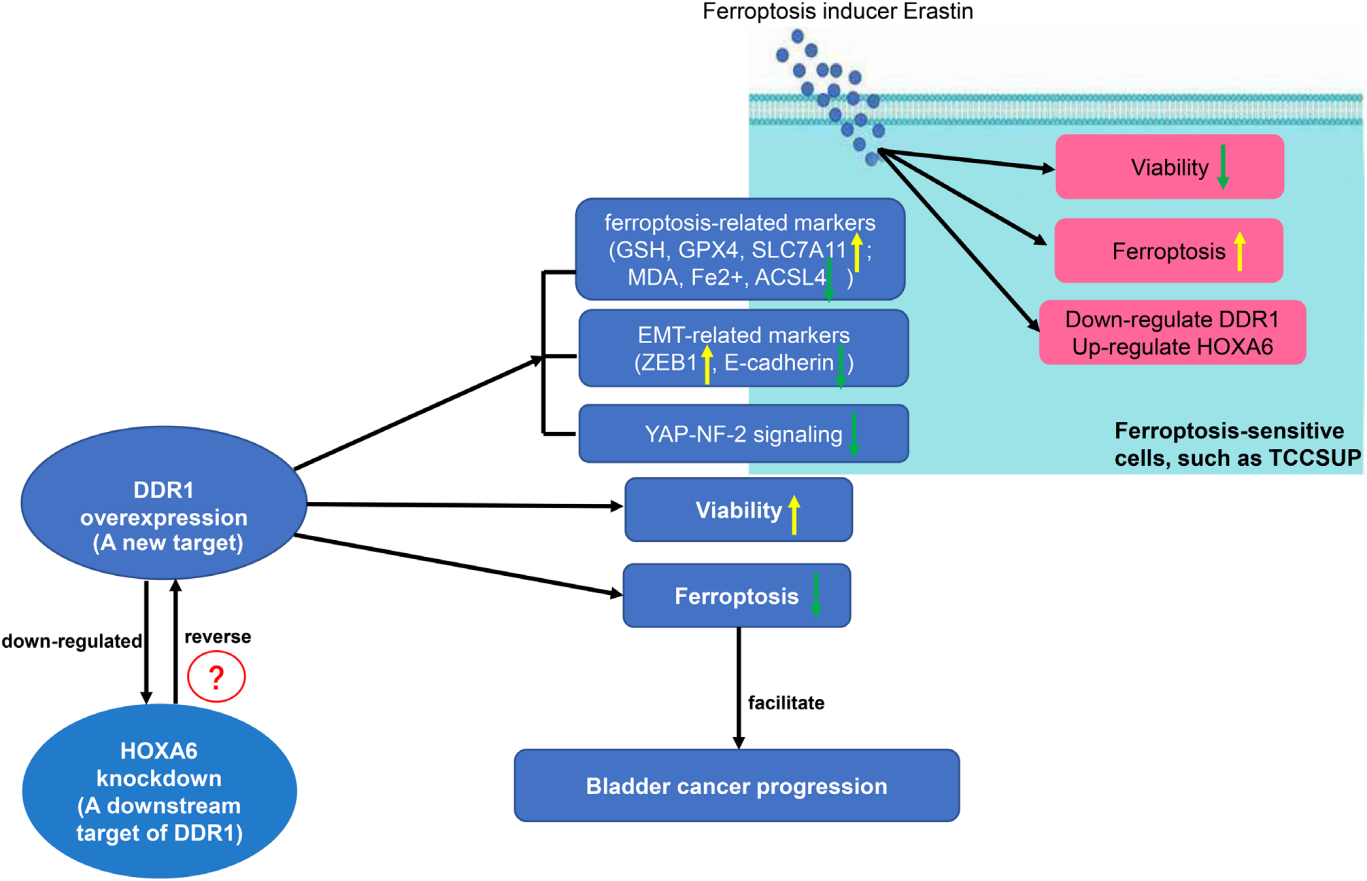
Schematic diagram of our study. The ferroptosis inducer erastin inhibited the viability of ferroptosis‐sensitive TCCSUP cells, promoted ferroptosis and downregulated DDR1 while upregulating HOXA6. Additionally, DDR1 overexpression regulated ferroptosis‐related markers (upregulation of GSH, GPX4 and SLC7A11 expression, and down‐regulation of MDA, Fe^2+^ and ACSL4 levels) and EMC‐related markers (downregulation of E‐cadherin and upregulation of ZEB1), and inhibited YAP–NF‐2 signalling pathway activation, promoting the viability of TCCSUP cells and inhibiting ferroptosis, thereby facilitating bladder cancer progression. HOXA6 may be a downstream target of DDR1, and DDR1 may negatively regulate HOXA6. HOXA6 knockdown reversed the effects of DDR1 overexpression on ferroptosis in TCCSUP cells. Yellow arrow: upregulation or promotion, green arrow: downregulation or inhibition and ?: there may be other underlying mechanisms between the two.

In summary, we found that DDR1, an important inhibitor of BC ferroptosis, may inhibit BC cell ferroptosis and promote BC progression by targeting HOXA6 (Figure 9). This study sheds light on the important role of DDR1 in BC ferroptosis and suggests that DDR1 may serve as a potential target for BC diagnosis and treatment.

## Author Contributions

**Xin Xie:** conceptualization (equal), investigation (equal), methodology (equal), writing – original draft (equal). **Hongchao He:** formal analysis (equal), investigation (equal), methodology (equal), validation (equal). **Ning Zhang:** formal analysis (equal), investigation (equal), software (equal), validation (equal). **Xiaojing Wang:** data curation (equal), formal analysis (equal), investigation (equal), software (equal). **Wenbin Rui:** formal analysis (equal), investigation (equal), methodology (equal), software (equal). **Danfeng Xu:** investigation (supporting), methodology (supporting), software (equal), validation (supporting). **Yu Zhu:** investigation (supporting), methodology (supporting), resources (equal), software (supporting), validation (supporting). **Ming Tian:** conceptualization (supporting), funding acquisition (supporting), project administration (equal), supervision (supporting), writing – review and editing (supporting). **Wei He:** conceptualization (lead), funding acquisition (equal), project administration (equal), supervision (equal), writing – review and editing (equal).

## Ethics Statement

This study was approved by the Animal Experimental Ethics Committee of Ruijin Hospital, Shanghai Jiao Tong University School of Medicine.

## Consent

The authors have nothing to report.

## Conflicts of Interest

The authors declare no conflicts of interest.

## Supporting information


Figure S1.

## Data Availability

The dataset used and/or analysed during the current study are available from the corresponding author on a reasonable request.
