# Supplementary material for: DDR1 Targeting HOXA6 Facilitates Bladder Cancer Progression via Inhibiting Ferroptosis
Source: J Cell Mol Med. 2025 Mar 19;29(6):e70410. doi: 10.1111/jcmm.70410 (PMC11921465; doi:10.1111/jcmm.70410)
Supplement: Supplementary file 1 — Figure S1. [file JCMM-29-e70410-s001.doc]

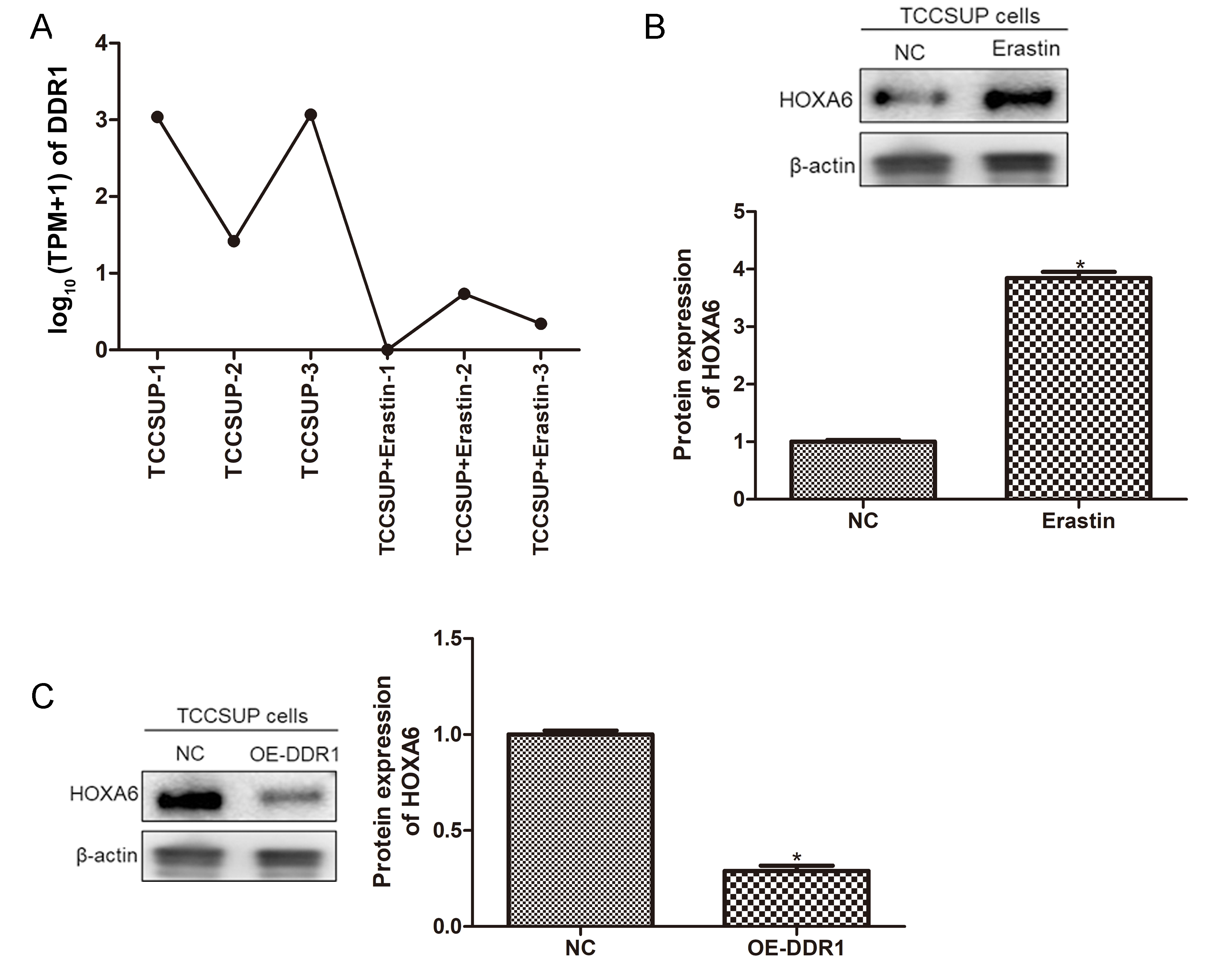


Supplementary figure 1 The expression of DDR1 and HOXA6. (A) The expression of DDR1 in each sample through sequencing. (B) The protein expression of HOXA6 in the control and erastin-treated TCCSUP cells using western blot. * *P* < 0.05 versus Control group. (C) The protein expression of HOXA6 in the control and DDR1-overexpressed TCCSUP cells using western blot. * *P* < 0.05 versus Control group.
